# Enhancer of zeste acts as a major developmental regulator of *Ciona intestinalis* embryogenesis

**DOI:** 10.1242/bio.010835

**Published:** 2015-08-14

**Authors:** Emilie Le Goff, Camille Martinand-Mari, Marianne Martin, Jérôme Feuillard, Yvan Boublik, Nelly Godefroy, Paul Mangeat, Stephen Baghdiguian, Giacomo Cavalli

**Affiliations:** 1 Université Montpellier, Place Eugène Bataillon, Montpellier 34095, Cedex 5, France; 2 Institut des Sciences de l'Evolution (ISEM), UMR5554, CNRS, Montpellier 34095, France; 3 Dynamique des interactions membranaires normales et pathologiques (DIMNP), UMR 5235, CNRS, Montpellier 34095, France; 4 Centre de Recherche de Biochimie Macromoléculaire (CRBM), UMR5237, CNRS, Montpellier 34293, Cedex 05, France; 5 Institute of Human Genetics (IGH), UPR 1142, CNRS, Montpellier 34396, France

**Keywords:** *Ciona intestinalis*, Enhancer of zeste, Polycomb, PRC2, Embryogenesis

## Abstract

The paradigm of developmental regulation by Polycomb group (PcG) proteins posits that they maintain silencing outside the spatial expression domains of their target genes, particularly of *Hox* genes, starting from mid embryogenesis. The Enhancer of zeste [E(z)] PcG protein is the catalytic subunit of the PRC2 complex, which silences its targets via deposition of the H3K27me3 mark. Here, we studied the ascidian *Ciona intestinalis* counterpart of E(z). Ci-E(z) is detected by immunohistochemistry as soon as the 2- and 4-cell stages as a cytoplasmic form and becomes exclusively nuclear thereafter, whereas the H3K27me3 mark is detected starting from the gastrula stage and later. Morpholino invalidation of Ci-E(z) leads to the total disappearance of both Ci-E(z) protein and its H3K27me3 mark. Ci-E(z) morphants display a severe phenotype. Strikingly, the earliest defects occur at the 4-cell stage with the dysregulation of cell positioning and mitotic impairment. At later stages, Ci-E(z)-deficient embryos are affected by terminal differentiation defects of neural, epidermal and muscle tissues, by the failure to form a notochord and by the absence of caudal nerve. These major phenotypic defects are specifically rescued by injection of a morpholino-resistant Ci-E(z) mRNA, which restores expression of Ci-E(z) protein and re-deposition of the H3K27me3 mark. As observed by qPCR analyses, Ci-E(z) invalidation leads to the early derepression of tissue-specific developmental genes, whereas late-acting developmental genes are generally down-regulated. Altogether, our results suggest that Ci-E(z) plays a major role during embryonic development in *Ciona intestinalis* by silencing early-acting developmental genes in a *Hox*-independent manner.

## INTRODUCTION

Epigenetic regulation of gene expression is necessary for the correct processing of developmental programs and the maintenance of cell fates. Polycomb group (PcG) and Trithorax group (TrxG) genes were first identified genetically in *Drosophila melanogaster* as respective repressors and activators required for maintaining the proper expression pattern of homeotic genes (*Hox* genes) throughout development. The products of *Hox* genes, a set of transcription factors, specify cell identity along the antero-posterior axis of segmented animals. In addition to these developmental functions, PcG and TrxG proteins play critical roles in stem cell biology and are involved in pathological deregulations leading to cancer (Martinez et al., 2009; Sauvageau and Sauvageau, 2010; Simon and Kingston, 2009; Sparmann and van Lohuizen, 2006).

In Drosophila, three principal PcG protein complexes have been characterized: the Polycomb repressive complex 1 and 2 (PRC1 and PRC2, respectively) and the Pho repressive complex (PhoRC) (Lanzuolo and Orlando, 2012; Schwartz and Pirrotta, 2007). Enhancer of zeste, E(z), is one of the four major components of the PRC2 which also includes Extra sex comb (Esc), Suppressor of zeste 12 (Su(z)12) and Nurf-55. PRC2 is known to associate with and trimethylate nucleosomes specifically at Lysine 27 of histone H3 (H3K27me3 mark) via its catalytic SET domain (Cao and Zhang, 2004) which is activated when E(z) is associated with the three other PRC2 components (Czermin et al., 2002; Müller et al., 2002). H3K27 is also subjected to mono and di-methylation and these marks are also E(z) dependent (Ferrari et al., 2014). E(z) loss of function induces the lack of H3K27 methylation, implying that K27-specific methyltransferase activity is only supported by E(z) (Ebert et al., 2004). The H3K27me3 mark is associated with transcriptional repression and to the recruitment of the PRC1 complex, which consists of the core components Polycomb (Pc), Polyhomeotic (Ph), Posterior sex comb (Psc), and dRing (Aïssani and Bernardi, 1991; Müller and Verrijzer, 2009; Schuettengruber et al., 2007; Schwartz and Pirrotta, 2007; Simon and Kingston, 2009). PRC1 adds a second histone mark consisting in mono-ubiquitinylation of Lys119 on histone H2A, via the ubiquitin-ligase of dRing (Wang et al., 2004).

PcG proteins are generally considered as major epigenetic regulators of development in metazoans. In particular, PRC2 components are widely conserved in plants and animals, whereas the evolution of PRC1 is more complex, with an increase in PRC1 homologs due to subsequent duplications in vertebrates (Kerppola, 2009; Whitcomb et al., 2007) and a loss of some PRC1 proteins in some metazoan species (Schuettengruber et al., 2007). *Ciona intestinalis*, a solitary ascidian (Tunicata, Chordata) is part of the sister group of the vertebrates (Delsuc et al., 2006). Fertilized eggs develop into tadpole larvae, which present a prototypical morphogenesis and chordate body plan, characterized by the presence of a hollow dorsal neural tube, a notochord, paraxial mesoderm and a post-anal tail (Satoh, 1994, 2008). Beside a tadpole-like chordate body plan, the lineage of *Ciona intestinalis* embryonic cells is invariant and has been well described (Conklin, 1905; Lemaire, 2009). Its genome is fully sequenced and largely annotated (Dehal et al., 2002).

In *Ciona intestinalis*, the *Hox* gene cluster is disorganized and dispersed across two chromosomes; the temporal colinearity of *Hox* gene expression, classically described in other species, is lost and the spatial colinearity is only partially retained (Ikuta et al., 2004). The functional roles of *Hox* genes are limited, as far as larval development is concerned (Ikuta et al., 2010). Intriguingly, although PRC2 is fully present (Schuettengruber et al., 2007), *Ciona intestinalis* PRC1 apparently lacks the Pc subunit of PRC1 which recognizes the H3K27me3 mark deposited by PRC2, thus leaving open the question as to whether PRC1 is active in *Ciona intestinalis*. Most importantly, is Ci-E(z) functional in the absence of Pc? In this study, we analyzed the function of the Ci-E(z) homolog during embryonic development. In order to address this question, we used an invalidation approach by morpholinos injected in eggs before fertilization and followed Ci-E(z) protein expression and activity during embryo development.

## RESULTS

### Ci-E(z) has a specific developmental expression pattern and its inhibition causes major developmental defects

**Fig. 1.**
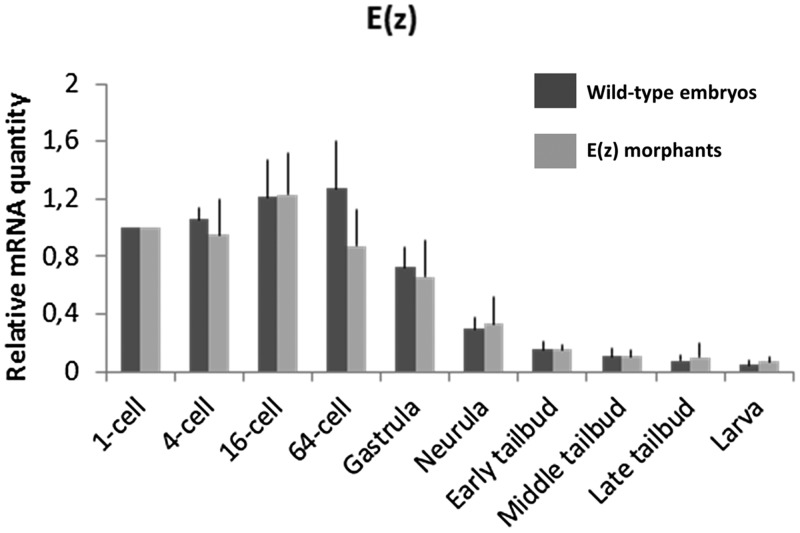
**mRNA expression of Ci-E(z) in wild-type embryos and Ci-E(z) morphants.** Time course of Ci-E(z) mRNA expression between 1-cell stage to hatching stage in Ci-E(z) morphants (light grey) and wild-type embryos (dark grey). Histograms are the mean of four independent micro-injection experiments; data were normalized to respective S26 mRNA expression values. The “relative mRNA quantity” was expressed with a value of 1 set as the amount of mRNA at 1-cell stage. No significant difference in mRNA levels was observed between wild-type and morphants at any stage. Error bars correspond to the standard deviation (s.d.) from four independent experiments.

*Ci-E(z)* gene is maternally expressed and its relative mRNA content is maximal at the 64-cell stage and decreases gradually over time (Fig. 1). In order to repress Ci-E(z) function, *Ciona intestinalis* eggs were injected with either Ci-E(z) or control morpholinos. Two Ci-E(z) morpholinos were designed with the aim to target the AUG codon and generate untranslatable mRNAs. Both morpholinos induced the same phenotype (data not shown), so only one (#1, see Materials and Methods) of them was used in further experiments. Following morpholino injection we verified, by qPCR, the level of mRNA expression of Ci-E(z) (Fig. 1). No significant difference between control and morphant embryos was observed, consistent with the fact that injection of Ci-E(z) morpholino should only induce a defective expression of the protein. In order to detect Ci-E(z) protein, we raised a specific antibody from a recombinant N-terminal fragment of Ci-E(z).

**Fig. 2.**
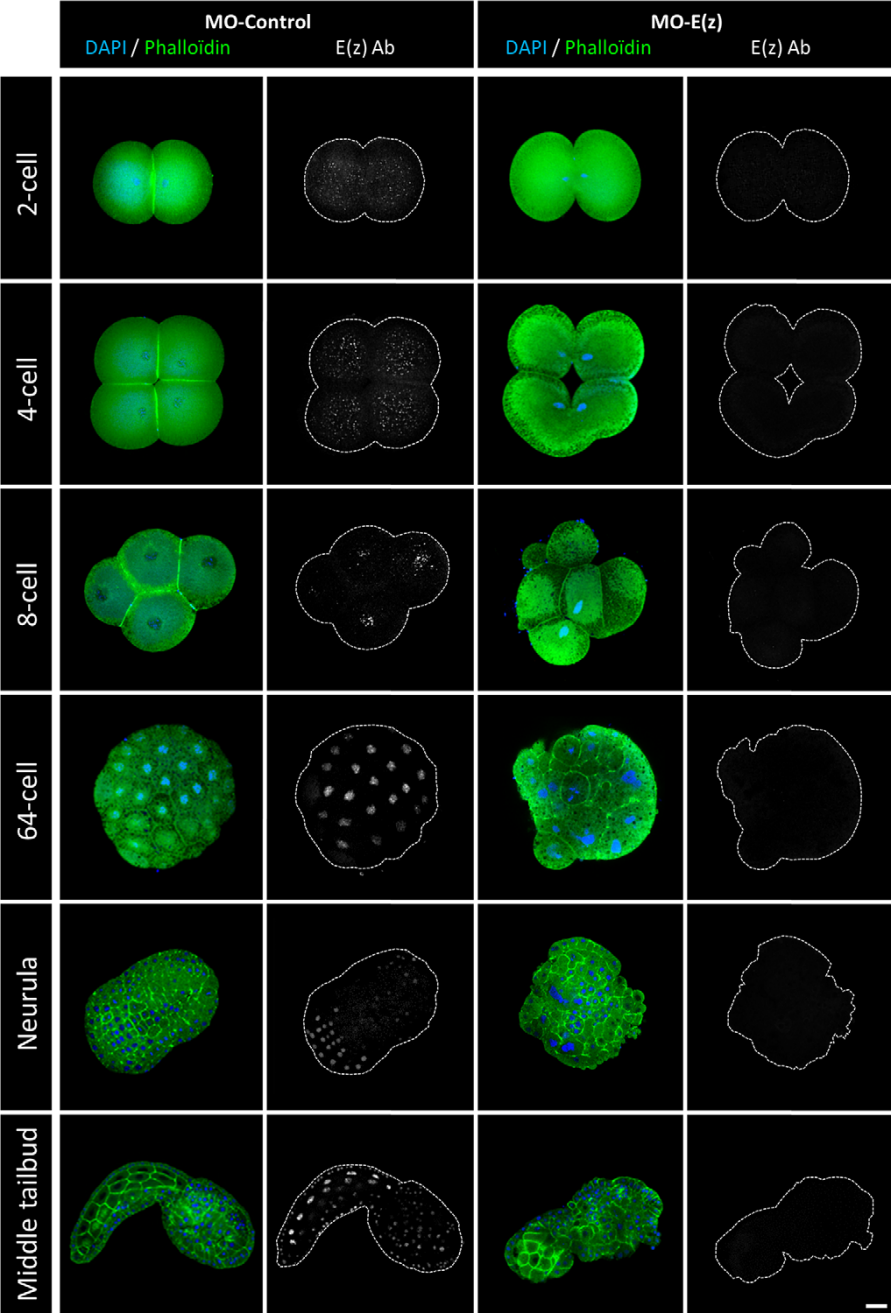
**Localization of Ci-E(z) protein in control embryos and Ci-E(z) morphants.** Ci-E(z) protein, actin (Phalloïdin, green) and DNA (DAPI, blue) were localized by triple labeling in *Ciona intestinalis* embryos at different stages of development by confocal microscopy. At the right of each merge (actin/DNA) the corresponding Ci-E(z) image is shown with the cell contours drawn in grey. For the 64-cell and neurula wild-type stages, the Ci-E(z) images correspond to a stacked image of all z planes. Each point of kinetic was repeated between 4 and 10 times. For each kinetic point, between 20 and 40 embryos were collected. Scale bar: 25 µm.

The expression and cellular localization of Ci-E(z) protein during embryonic development of *Ciona intestinalis* was next characterized in control and Ci-E(z) morphants (Fig. 2). In control morphants, Ci-E(z) protein was detected as early as the 2-cell stage and throughout all stages of larva embryogenesis (Fig. 2, left panels and data not shown). At the 2- and 4-cell stages, E(z) protein was predominantly detected in the cytoplasm of all blastomeres. In contrast, in the further developmental stages, E(z) was only detected in the nucleus, with all blastomeres being labeled until the 64-cell stage (except when cells underwent mitosis, which induced loss of chromosomal Ci-E(z) staining). At neurula stage, the labeling was detected in the majority of cells from the future posterior part of larva. In the subsequent stage of development (initial tailbud), Ci-E(z) protein expression was mainly muscle and epidermis specific and, at middle tailbud stage, the fluorescence intensity became maximal with Ci-E(z) being detected in all embryonic cells. In late tailbud, when the notochord development is achieved, all tail cells were Ci-E(z) positive (supplementary material Fig. S1). At hatching, a new differential expression of Ci-E(z) was observed. Cells of the newly formed adhesive papillae were Ci-E(z)-positive, as well as some epidermal and endodermal head cells. In the tail, positive cells were specifically located in the extremity including epidermis, muscle and notochord cells (supplementary material Fig. S1).

We next analyzed the effect of Ci-E(z) morpholino injection. The invalidation of Ci-E(z) protein expression was effective since, in contrast to control embryos (Fig. 2, left panels), Ci-E(z) immunofluorescence became undetectable during embryonic development (Fig. 2, right panels and data not shown). Ci-E(z) morphants exhibited major developmental defects: blastomere division was impaired, leading to the development of a disorganized embryo. Indeed as early as at the 4-cell stage, a significant defect in cell positioning took place. This embryo asymmetry was amplified during subsequent divisions, which led to the formation of disorganized, asymmetric gastrula and neurula embryos. The morphant embryo failed to reach a functional hatching larva, leading to an impaired tadpole in which a head structure could still be distinguished from an incomplete tail part totally lacking the notochord structure. Features of cell differentiation were observed, but the differentiation program was incomplete. For instance, muscle-like cells could be identified (Fig. 2: stronger Phalloïdin staining at middle tailbud stage). Throughout the development, multinucleated cells were also observed confirming a cell division defect (supplementary material Fig. S2). At the latest stage, rare Ci-E(z) positive cells appeared, potentially indicative of the occurrence of a dilution effect of the morpholino. It is important to note that, at any stage of embryonic development, no programmed cell death occurred as revealed by the lack of TUNEL staining in Ci-E(z) morphants (data not shown).

### Ci-E(z) is required for the correct division and differentiation of all major developmental cell types

**Fig. 3.**
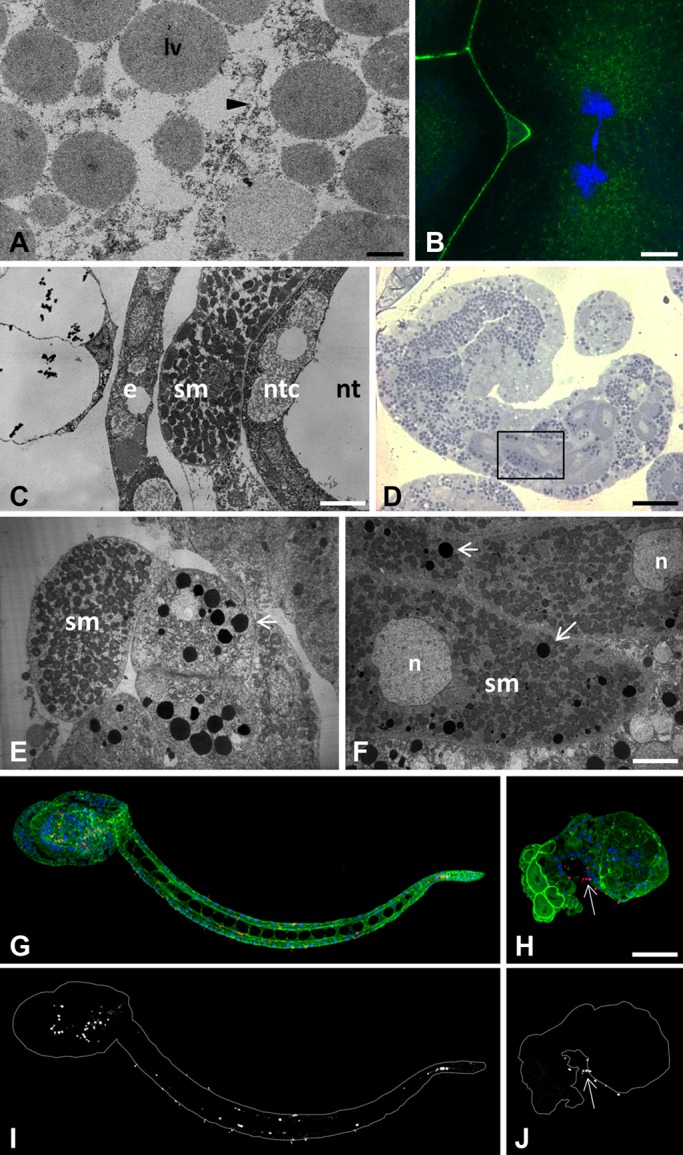
**Characterization of major phenotypic defects induced by Ci-E(z) invalidation at the 4-cell and hatching larva stages.** (A,B) Ci-E(z) morphants are affected by cytokinesis defect at the 4-cell stage as demonstrated by TEM (A) and indirect immunofluorescence (B) analyses. The black arrowhead in A points to incomplete cell membrane formation. lv, lipid vesicle. (B) Actin (Phalloïdin, green) and DNA (DAPI, blue) double labeling. (C-F) Ultrastructural characterization of wild type (C) hatching larvae and Ci-E(z) morphants (D-F). (D) Semi-thin section of a Ci-E(z) morphant, stained with Toluidine Blue. The black rectangle window corresponds to the enlarged ultra-thin section TEM image shown in (F), showing abnormal accumulation of lipid vesicles (arrows) in muscle cells. Ultra-thin tail cross-sections of wild-type hatching larva (C) and Ci-E(z) morphant (E) showing the externally mislocalized muscle cell in Ci-E(z) morphants. e, epiderm cell; sm, striated muscle; ntc, notochord cell; nt, notochord lumina; n, nucleus. (G-J) Localization of neuronal form of tubulin in hatching larvae from control (G) and Ci-E(z) morphant (H). Acetylated-tubulin (red), actin (Phalloïdin, green) and DNA (DAPI, blue) triple labeling of *Ciona intestinalis* hatching larvae (G,H). Respective corresponding images (I,J) of acetylated-tubulin alone are shown in grey. The arrow indicates the position of nervous cells at the tail-head junction. Scale bars: 10 µm (C); 35 µm (D); 6 µm (F); 2 µm (A); 5 µm (B); 70 µm (H).

The characterization of the Ci-E(z)-invalidated phenotype was further analyzed at 4-cell and hatching larval stages (Fig. 3). An extensive comparison between control and Ci-E(z) morphant embryos was performed. First, cells were characterized and identified at the ultrastructural level. In ultrathin sections, at the 4-cell stage, defects in cytokinesis with incomplete cell membrane formation (Fig. 3A), leading to multinucleated blastomeric cells (seen by immunofluorescence, Fig. 3B), were the striking earliest features of the Ci-E(z)-morphant phenotype. At the hatching larval stage, in contrast to control embryos (Fig. 3C), the relative positioning of cells was drastically affected in the morphant (Fig. 3E): the lack of notochord structure was particularly striking, pointing to the disorganization of embryonic tissues. In addition to the lack of notochord formation, a major feature of Ci-E(z) invalidation appeared to be a general defect in cell differentiation, which led to the generation of a majority of cells abnormally rich in lipid droplets, as evidenced by the toluidin blue staining in semi-thin sections of morphant cells (Fig. 3D). Mitochondria-enriched muscle cells were clearly identified as being externally delocalized compared with control tissue. These muscle cells were found located next to a set of undifferentiated cells containing numerous lipid droplets, presumably originating from endodermal precursor cells (Fig. 3E). Muscle cells appeared abnormally rich in lipid droplets as well (Fig. 3F). Last, a specific-antibody (acetylated-tubulin) was used to identify differentiated neural cells at the hatching larval stage. In control embryos, acetylated-tubulin stained cells helped specifying the localization of both the central (head-located part) and peripheral (regularly positioned all along the tail) nervous systems (Fig. 3G,I). In contrast, Ci-E(z) morphants displayed very few neural cells that were mislocalized at the head-tail junction (Fig. 3H,J). Moreover, the caudal nerve cord was totally lacking.

### Ci-E(z) is responsible for H3K27me3 mark deposition

**Fig. 4.**
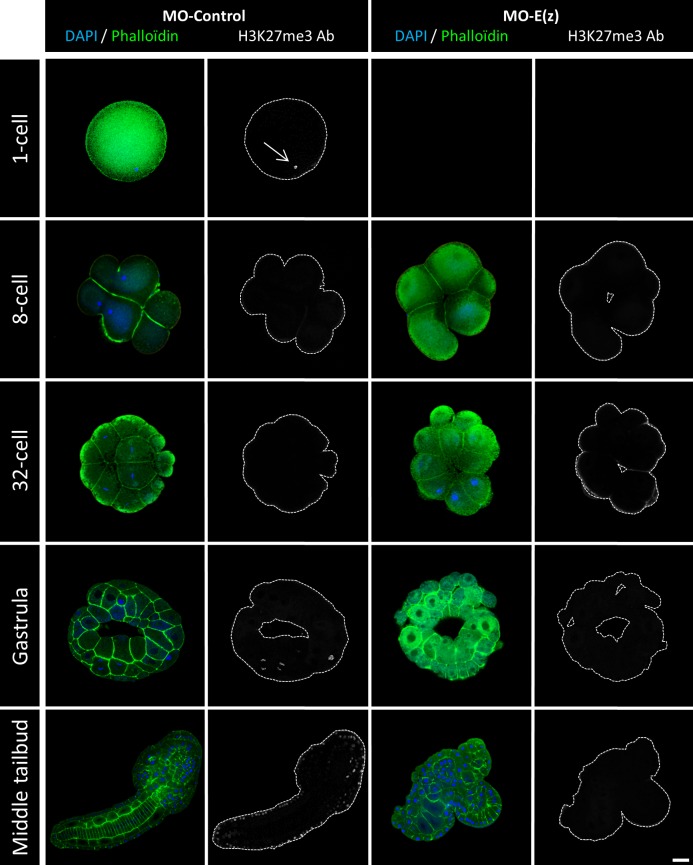
**H3K27me3 detection by indirect immunofluorescence in control embryos and Ci-E(z) morphants.** H3K27me3, actin (Phalloïdin, green) and nuclei (DAPI, blue) triple labeling of *Ciona intestinalis* embryos at different stages of development and confocal analyses. On the right of each merge, the corresponding image of H3K27me3 staining is shown with the cell contour drawn in grey. Each point of kinetic was repeated between 4 and 10 times. For each kinetic point, between 20 and 40 embryos were collected. The arrow shows H3K27me3 detection in the pronucleus. Scale bar: 25 µm.

**Fig. 5.**
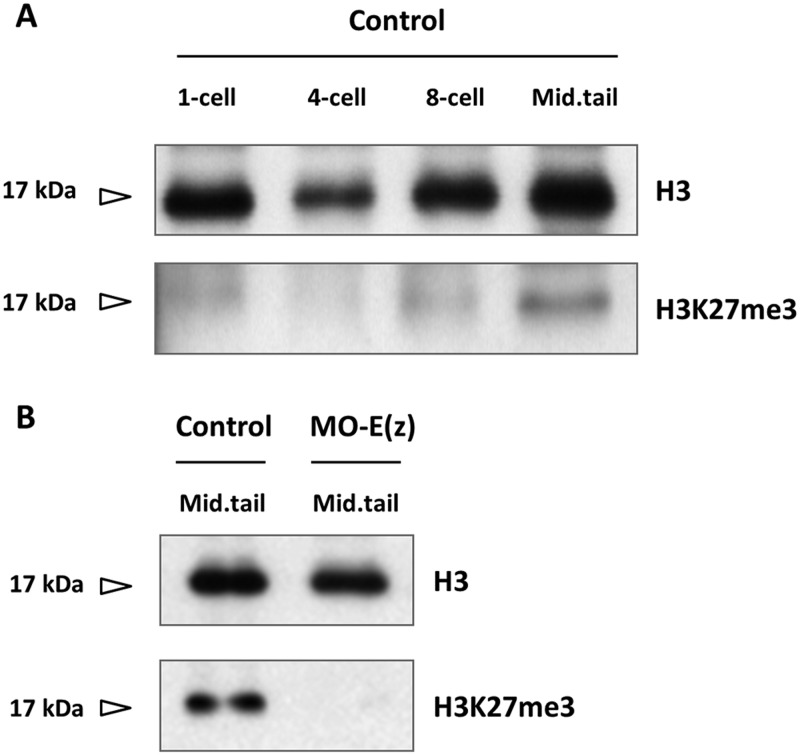
**H3K27me3 detection by western blotting in control embryos and Ci-E(z) morphants.** (A) Control embryos (MO-control) extracts from different stages of *Ciona intestinalis* were western blotted with antibodies directed specifically against histone H3 (H3) and the trimethylated form of H3 (H3K27me3). Mid.tail, middle tailbud stage. (B) Comparative western blot analysis between control embryos (MO-control) and Ci-E(z) morphants [MO-E(z)] at middle tailbud stage of *Ciona intestinalis.* Extracts were treated as in A.

Within the PRC2 complex, Ci-E(z) bears the catalytic activity responsible for the deposition of the H3K27me3 mark (Ferrari et al., 2014). It was therefore expected that the invalidation of Ci-E(z) should result in the lack of H3K27 trimethylation. We verified the status of the H3K27me3 mark with a specific antibody in control and Ci-E(z) morphants during embryonic development (Fig. 4 and data not shown). Interestingly, using immunofluorescence staining, the H3K27me3 antibody labeled the egg pronucleus at meiosis 1 stage (Fig. 4, left panels) whereas the Ci-E(z) protein was undetectable. From 2-cell up to 64-cell stage, the staining for the mark was negative in control embryos, although Ci-E(z) was detected as described above (Fig. 2, left panels). In gastrula, the H3K27me3 mark reappeared and the labeling was maintained in neurula. In the initial tailbud stage, only epidermal cells of the nascent tail were H3K27me3 positive. A strong labeling was then observed at middle tailbud stage by immunofluorescence staining (Fig. 4, left panels), and correlated with the strongest detection of Ci-E(z) protein (Fig. 2, left panels), in some endodermal cells of the head and in some cells from epidermal origin and of the tail extremity. A similar pattern of labeling was maintained in later stages. In the hatching larva, the cells of papillae were H3K27me3 positive, as was previously observed for Ci-E(z) (supplementary material Fig. S1). In summary, the H3K27me3 mark appeared after a lag phase during early embryogenesis, in which Ci-E(z) was detected without apparent H3K27me3, and then matched Ci-E(z) distribution throughout development. Of note H3K27me3 was maintained during mitosis (supplementary material Fig. S3), consistent with a relatively slow histone turnover and demethylation rate. Importantly, no detection of the H3K27me3 mark was observed in Ci-e(z) morphants (Fig. 4 right panels), demonstrating that Ci-E(z) is the only histone K27-specific methyltransferase, as already observed in humans (Ferrari et al., 2014). For clarity, Table 1 recapitulates the immunofluorescence detections of respectively Ci-E(z) and H3K27me3 mark throughout all stages of embryogenesis. As in control kinetics, the mark was only revealed at gastrula stage although the nuclear presence of Ci-E(z) was detected as soon as the 8-cell stage, and preceded by an earliest cytoplasmic form. Immunofluorescence results were controlled with western blotting analyses (Fig. 5A). In control embryos, the H3K27me3 mark was faintly detected at the 1-cell stage, then dropped at 4-cell stage and became weakly detectable at 8-cell stage (suggesting that, although not seen in immunofluorescence, initial H3K27 trimethylation may be occurring at this stage) and significantly stronger in middle tailbud. In sharp contrast to the wild type condition, the lack of H3K27me3 mark in the morphant context was clearly confirmed (Fig. 5B). Therefore, all results converge to show that Ci-E(z) is responsible for all H3K27me3 trimethylation during *Ciona intestinalis* embryogenesis.

**Table 1.**
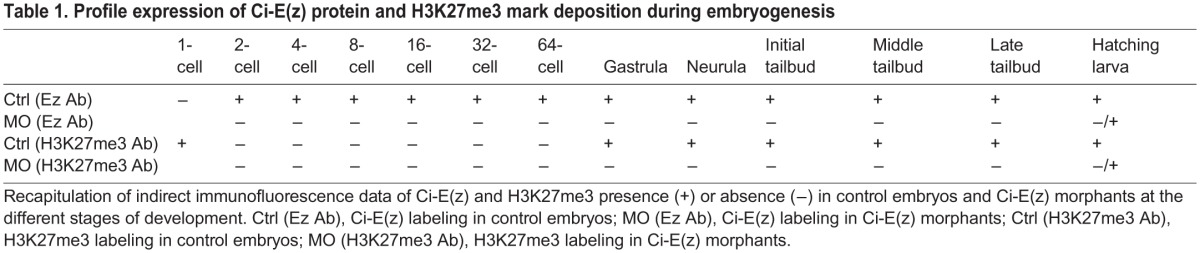
**Profile expression of Ci-E(z) protein and H3K27me3 mark deposition during embryogenesis**

### The invalidation of Ci-E(z) can be specifically rescued

**Fig. 6.**
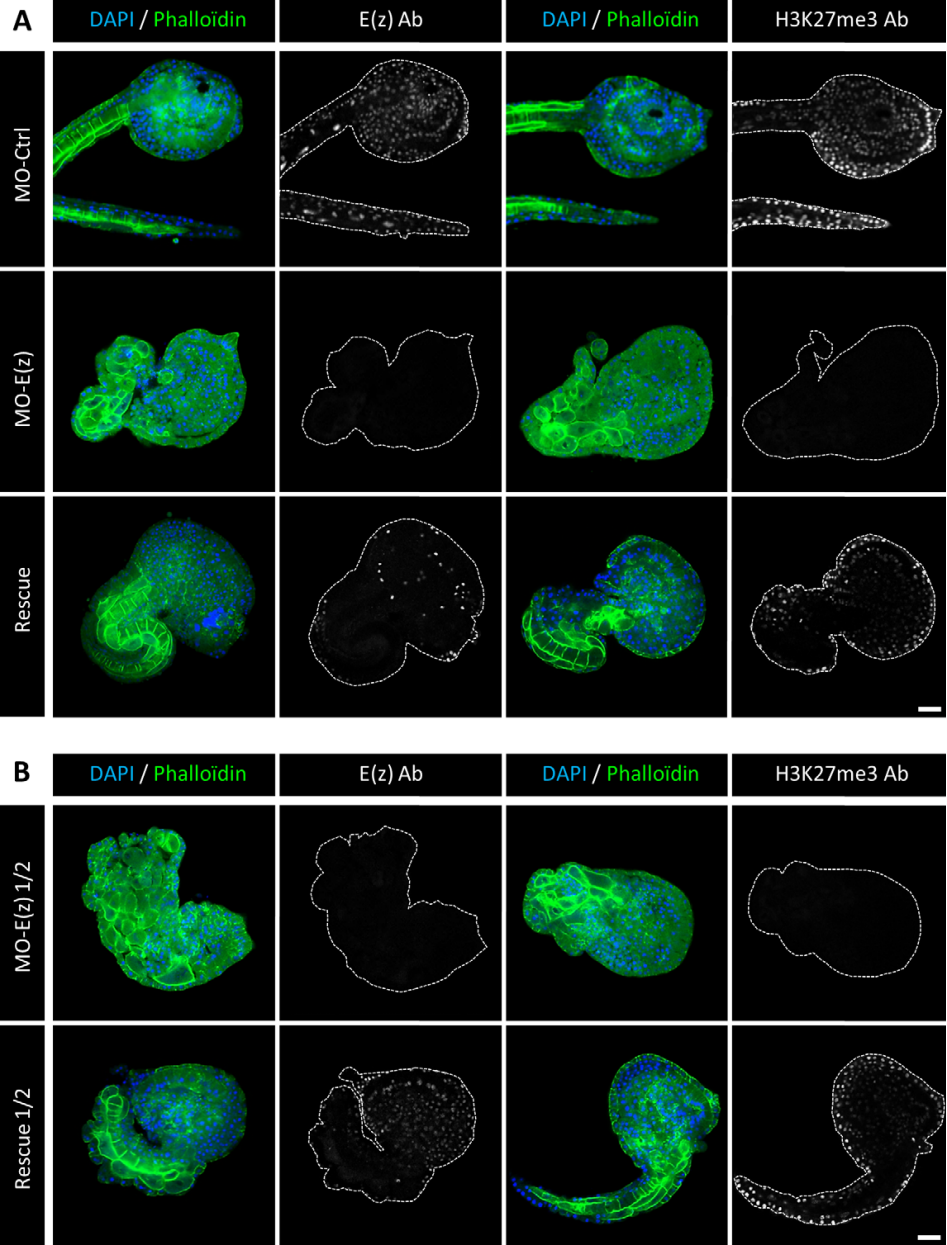
**Ci-E(z) and H3K27me3 detection in rescue experiments.** Comparative indirect immunofluorescence analysis between (A) control embryos, Ci-E(z) morphants, rescue embryos and (B) Ci-E(z) morphants ½ and rescue ½ embryos at late tailbud stage of development. A triple labeling was performed using: actin (Phalloïdin in green), DNA (DAPI, in blue) and antibodies against H3K27me3 or Ci-E(z) protein (in grey). On the right of each DAPI/Phalloïdin merge, the corresponding image of Ci-E(z) or H3K27me3 alone is shown (as indicated) with cell contour drawn in grey. Ab, antibodies; MO-Ctrl, control embryo; mRNA-E(z), mRNA-E(z) embryo; MO-E(z), Ci-E(z) morphant; MO-E(z) ½, embryo injected with half of MO-E(z) concentration; Rescue, mRNA-E(z)+MO-E(z) embryo; Rescue ½, embryo injected with half of MO-E(z) concentration+mRNA-E(z). For three independent experiments, between 20 and 40 embryos were collected. Scale bar: 25 µm.

In order to confirm the specificity of Ci-E(z) morpholino, we tested whether the Ci-E(z) phenotype could be rescued by injection of a synthetic Ci-E(z) mRNA lacking the morpholino target sequence simultaneously with the Ci-E(z) morpholino. We first tested the MO-E(z)/mRNA-E(z) ratio classically used in *Ciona intestinalis* publications (Christiaen et al., 2009). Fig. 6A shows the comparison between control, E(z) morphant and rescue phenotypes obtained at late-tailbud stage. The control injection of mRNA-E(z) alone has no phenotypic effect (supplementary material Fig. S4). The rescued embryos presented partial restoration of E(z) protein levels as well as H3K27me3 mark labeling. At a morphological level, even though rescued embryos exhibited some disorganization features, most rescued embryos expressed a partially differentiated notochord that could be clearly identified by the coherent alignment of notochord cells at the tail-head junction (Fig. 6A, right rescue panels).

In a second set of rescue assays, and in order to improve the rescue, we used half the concentration of MO-E(z). Under these conditions, the morphant phenotype obtained was undistinguishable to the one obtained at the classical MO-E(z) concentration (Fig. 6B, MO-E(z) ½ panels). However, the rescued embryos were much more similar to the control phenotype (Fig. 6B, rescue ½ panels): restored E(z) protein expression as well as H3K27me3 mark labeling were more prominent. Importantly, the tail of rescued embryos expressed an almost complete notochord.

**Fig. 7.**
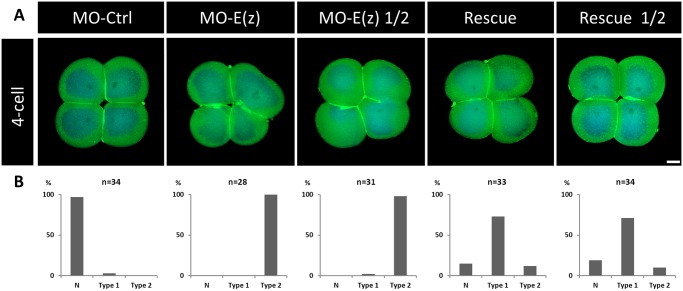
**Phenotypes obtained in rescue experiments at the 4-cell stage.** (A) Comparative indirect immunofluorescence analysis between control embryos, Ci-E(z) morphants, Ci-E(z) morphants injected with half of the MO-E(z) concentration, rescue embryos and rescue ½ embryos at 4-cell stage with a double labeling: actin (Phalloïdin, green) and DNA (DAPI, blue). MO-Ctrl, control embryo; MO-E(z), Ci-E(z) morphant; MO-E(z) ½, morphant injected with half of MO-E(z) concentration; Rescue, mRNA-E(z)+MO-E(z) embryo; Rescue ½, embryo injected with half of MO-E(z) concentration+mRNA-E(z). For each point, between 20 and 40 embryos were collected. Scale bar: 25 µm. (B) Corresponding percentage of embryos categorized as normal (N) or types 1–2. Type 1: Blastomeres are distinguishable from each other, embryo symmetry is not preserved. Type 2: Blastomeres are not properly separated, embryo symmetry is lost.

The results being striking at the larval stage, is the rescue active as soon as at the 4-cell stage? To answer this question, we performed rescue assays at this earlier stage when the E(z) inhibition is already effective (Figs 2 and 7). While both concentrations of MO-E(z) caused cytokinesis defects, co-injections of MO-E(z) with mRNA-E(z) restored a control phenotype with, notably, the rescue of the characteristic 4-cell symmetrical organization (Fig. 7, rescue and rescue ½ panels). These data show that Ci-E(z) mRNA injection does partially rescue both early and late effects of its impairment by morpholino, further substantiating the early and crucial role of this protein in *Ciona intestinalis* embryogenesis.

### Loss of Ci-E(z) induces specific de-repression of major developmental genes

**Fig. 8:**
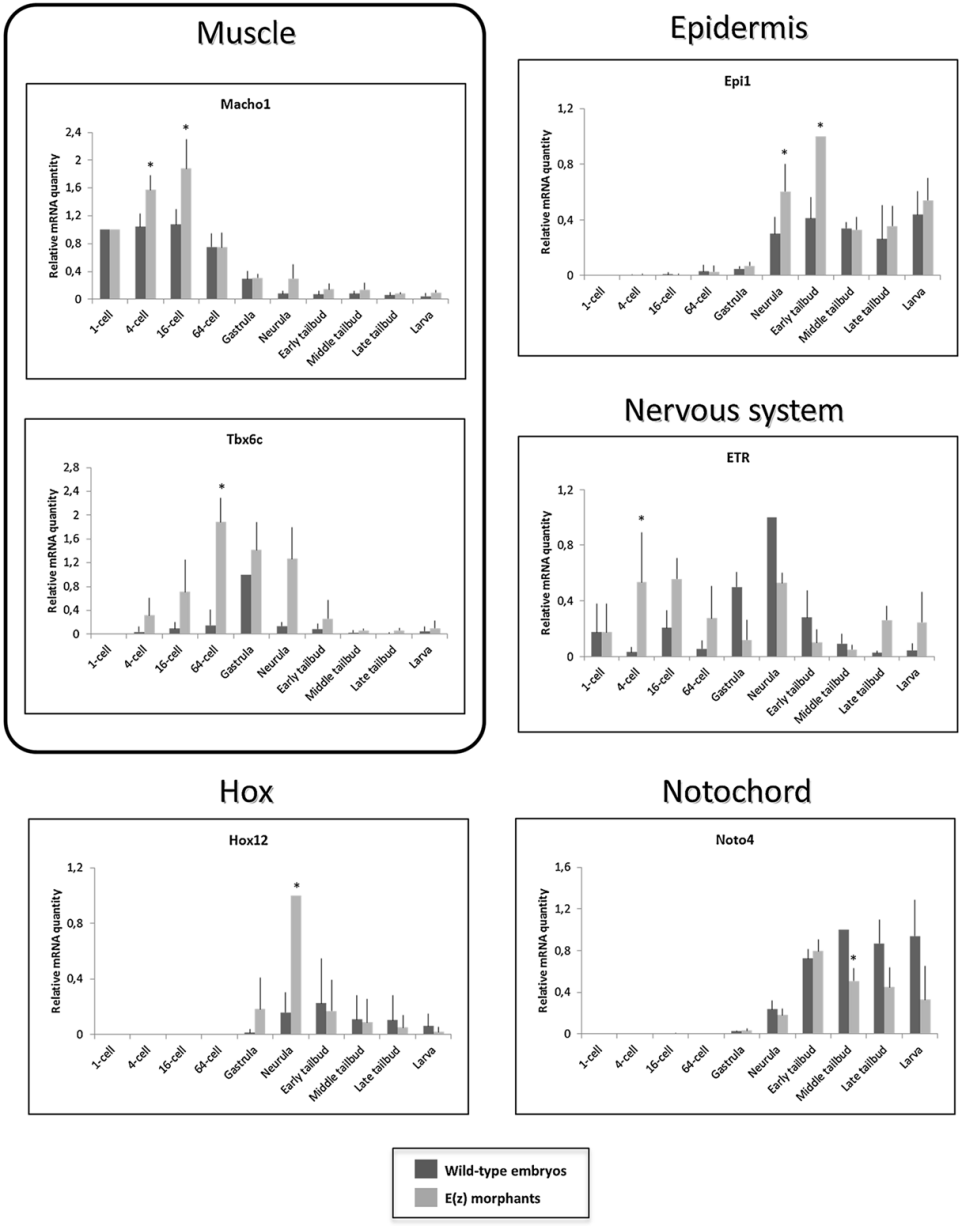
**. mRNA expression of tissue-specific genes and *Hox*12 gene in wild-type embryos and Ci-E(z) morphants.** Time course of muscle (*Macho1*, *Tbx6c*), nervous system (*ETR*), notochord (*Noto4*), epiderm (*Epi1*) specific and *Hox*12 mRNA expression between 1-cell to hatching stage in wild-type embryos (dark grey) and Ci-E(z) morphants (light grey). The histograms are the mean of four independent micro-injection experiments; data were normalized to respective S26 mRNA expression values. A relative mRNA quantity value of one corresponds to the highest amount of wild-type target mRNA except for *Hox*12 and *Epi1* mRNA expression for which it corresponds to the highest amount of Ci-E(z) morphant target mRNA. *P* values (*P<0.05) were calculated using the Wilcoxon rank sum test. Error bars correspond to the standard deviation from four independent experiments.

To complement the description of the Ci-E(z) phenotype, we further analyzed by qPCR the expression of various genes known to be implicated in the formation of embryonic tissues and organs (Fig. 8). We chose genes involved in muscle (*Macho1* and *Tbx6c*), nervous system (*ETR*), notochord (*Noto4*) and epidermal (*Epi1*) development as well as *Hox12* gene, the only *Hox* gene whose its morpholino slightly modifies the embryo development phenotype (Ikuta et al., 2010). A first category encompasses three genes (*Macho1, Tbx6c* and *ETR*) whose expression was increased in morphant context, starting from the 4-cell stage. For *Macho1* gene, its overall levels of expression declined in later development but with the significant maintenance of a relative difference between control and morphant. *Tbx6c*, a *Macho1*-induced gene (Yagi et al., 2005), was also overexpressed all along embryonic development up to hatching larval stage. In contrast, *ETR* overexpression was biphasic with a first peak up to 64-cell stage followed by down regulation at gastrula and a second phase of overexpression starting at late tailbud stage. It should be noticed that, in controls, the *ETR* expression peak was delayed to gastrula and neurula stages.

A second class of genes, *Hox12* and *Epi1*, was characterized by a peak of overexpression at neurula and early tailbud stages respectively. Finally, a last class of genes involved in terminal differentiation was found to be down regulated at the latest stages of embryonic development. *Noto4*, a gene involved in notochord formation, was significantly downregulated from middle tailbud stage and beyond.

**Fig. 9.**
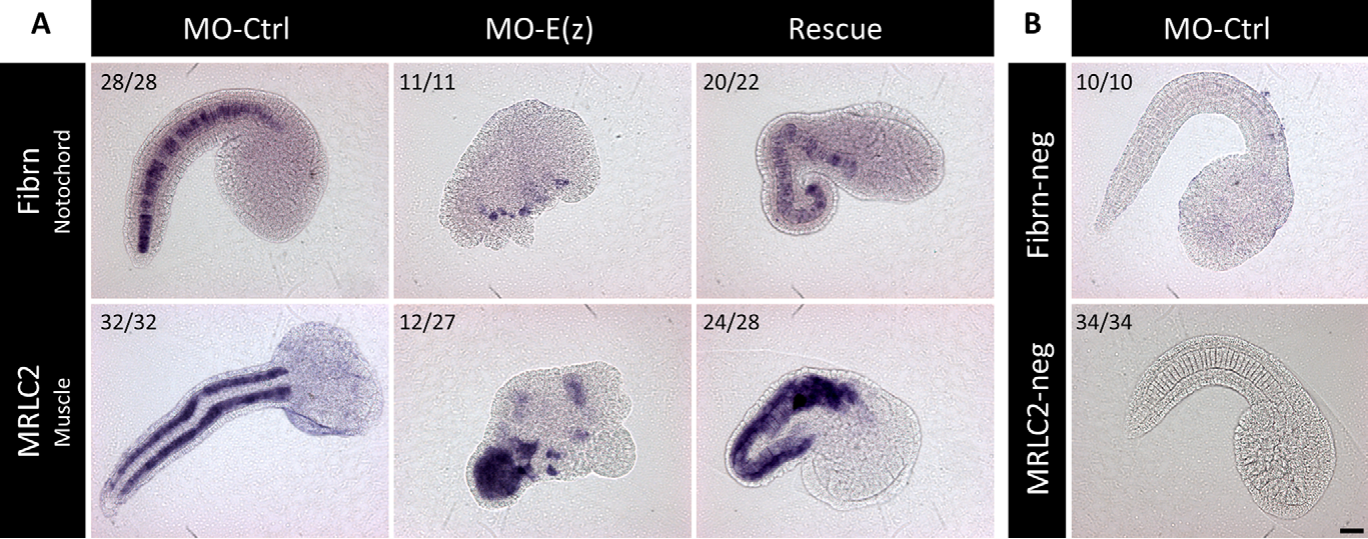
**Localization of tissue-specific markers in Ci-E(z) morphants, control and rescue embryos.** (A) Comparative *in situ* hybridization experiments were done with Ci-Fibrn (notochord specific) and Ci-MRLC2 (muscle specific) antisense probes at middle tailbud stage. MO-Ctrl, control embryo; MO-E(z), Ci-E(z) morphant; Rescue, mRNA-E(z)+MO-E(z) embryo. Number of embryos showing the presented expression pattern is indicated on each panel. For MRLC2, it is important to note that 27 of 27 morphants present muscle disorganization into the tail but only 12 of them have an additional ectopic expression in the head. (B) *In situ* hybridization negative controls done with Ci-Fibrn and Ci-MRLC2 sense probes. Scale bar: 30 µm.

Finally the strong phenotype observed in Ci-E(z) morphants was characterized further by *in situ* hydridization using two specific markers respectively of notochord and striated muscle, namely probes specific of Fibrinogen-like (Fibrn) (Takahashi et al., 1999) and Myosin Regulatory Light Chain 2 (MRLC2) (Ikuta et al., 2010). As observed in Fig. 9, Fibrn expression was found to be mostly absent in Ci-E(z) morphants consistent with the lack of notochord formation previously described (Figs 2, 3, 4 and 6). MRLC2 however was found highly expressed in a massive bulk of cells densely packed but highly disorganized, reminiscent of the mis-positioning characterized at the ultrastructural level (Fig. 3). It is interesting to note that out of 27 analyzed Ci-E(z) morphants, 12 of them expressed MRLC2 ectopically in some anterior localized cells. Through rescue one could detect that both the expression of Fibrn and MRLC2 was partially and significantly corrected (20 out 22 embryos for Fibrn and 24 out of 28 for MRLC2) (Fig. 9).

**Fig. 10:**
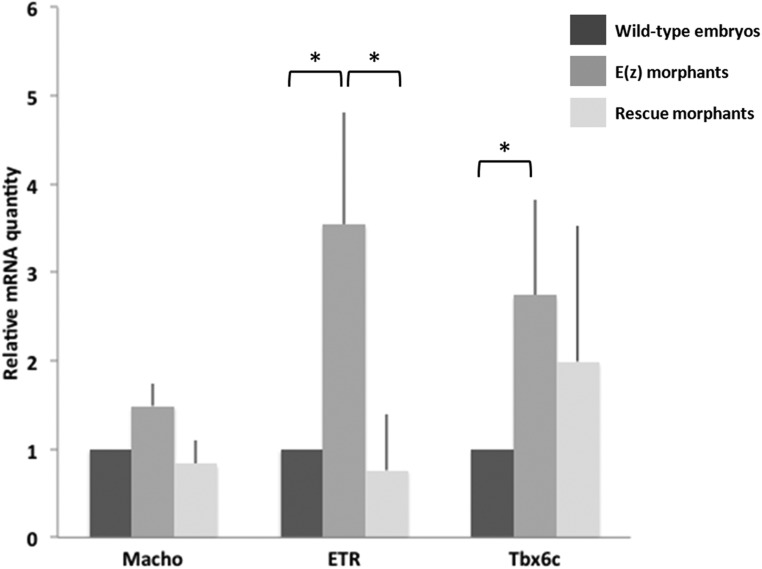
**. mRNA expression of tissue-specific genes in wild-type embryos, Ci-E(z) and Rescue morphants.** *Macho1*, *Tbx6c* and *ETR* mRNA expression at 16-cell stage in wild-type embryos (dark grey), Ci-E(z) morphants (intermediate grey) and Rescue embryos (light grey). The histograms are the mean of two (*Macho1*) or three (*Tbx6c* and *ETR*) independent micro-injection experiments; data were normalized to respective S26 mRNA expression values. A relative mRNA quantity value of one corresponds to the amount of wild type target mRNA. *P*-values (*P<0.05) were calculated using the Wilcoxon rank sum test. Error bars correspond to the standard deviation from three independent experiments.

In order to confirm the specificity of Ci-E(z) morpholino on genes presenting an early derepression (*Macho1, Tbx6c* and *ETR*), we tested by qPCR whether their 16-cell stage wild-type expression could be restored in the rescue experiment. As expected, the expression of the three genes was again suppressed, albeit with a lower efficiency for Tbx6c (Fig. 10).

Overall it can be concluded that the invalidation of Ci-E(z) through morpholino induces a strong phenotype (Figs 2 and 3) which correlates with derepression of target genes as early as at the 4-cell stage (Fig. 8), and which might be associated in part to the deficiency in H3K27me3 mark (Figs 4, 5 and 6) and to the deregulation of various sets of genes acting along the various stages of embryonic development, i.e. from very early on (4-cell stage) up to terminal differentiation stages (Figs 8, 9 and 10).

## DISCUSSION

The function of Polycomb proteins has not yet been reported in *Ciona intestinalis* and our data argue in favor of a major role for Ci-E(z) protein in the embryonic development of this organism. This conclusion is particularly noteworthy in the *Ciona intestinalis* context where the Pc protein which recognizes the H3K27me3 mark is absent. We showed that Ci-E(z) protein is present at all studied developmental stages with different localization and expression levels, mainly as a cytoplasmic form at 2- and 4-cell stages. The staining of the H3K27me3 mark is not detected before the gastrula stage, although weak H3K27me3 staining in western blot at the 8-cell stage suggests that some chromatin-specific PRC2 activity is present as early as its initial chromosome binding. Interestingly, in unfertilized eggs, H3K27me3 is present exclusively in the pronucleus; this latter observation, may be explained by the transient expression of Ci-Ez protein during the maturation of the egg in the ovarian tissue (data not shown) as previously described in Drosophila eggs where E(z) immunoreactivity was no longer detected at stage 6 of oogenesis, whereas the H3K27me3 mark was deposited earlier on and maintained throughout oogenesis (Iovino et al., 2013).

Invalidation of Ci-E(z) induces major defects in cellular positioning and differentiation, defects that could be restored in a rescue protocol. Ci-E(z) morphants lost their embryo symmetry as soon as 4-cell stage and were characterized by severe pleiotropic phenotypes, such as the absence of notochord and dorsal neural tube; positioning defects of muscle cells and epidermal sensory neurons; the presence of large multinucleated cells resulting from cytokinesis defects; and the perturbation of muscle cell metabolism with the accumulation of sarcoplasmic lipid vesicles (never seen in control muscle). This observation is reminiscent of the accumulation of lipid vesicles into cells that was reported during NAFLD (Non-Alcoholic Fatty Liver Disease), where the inhibition of EZH2 activity is associated with a lipid accumulation (Vella et al., 2013).

Since no abnormal apoptotic events occur at any stages of early embryonic development in Ci-E(z) morphants, the lack of notochord associated with the ectopic positioning of muscle and nerve cells are likely due to early cell fate defects. This is also in keeping with the fact that, at the 32-cell stage, the cell fate of the blastomeres is not fully committed (Satoh, 1994). For instance, the blastomere A6.4 pair shares a common notochord/muscle/nervous system destiny. B6.2 blastomeres possess a common notochord/muscle commitment, whereas A6.2 and B6.5 a common notochord/system nervous and nervous system/muscle cell fate respectively. At the 64-cell stage and the beginning of gastrulation, only blastomeres A7.8 and A8.16 share a common muscle/notochord destiny. In contrast, no such pluripotency is associated with blastomeres after the onset of gastrulation, suggesting that the effects of Ci-E(z) knock down results from cell fate problems arising earlier during development.

Strikingly, a morphant phenotype defect was observed as early as at the 4-cell stage. The Ci-E(z) mRNA being maternally provided, this suggests that some Ci-E(z) protein is required very early during embryonic development. Maternal to zygotic transition would seem to be an essential prerequisite for the proper processing of early embryonic cell divisions as soon as 2-cell stage like it was detected in mice embryos (Albert and Peters, 2009).

At the 4-cell stage, the inhibition of Ci-E(z) protein expression in morphants, which is mainly cytoplasmic, causes abnormal cell division with incomplete cell membrane formation. This phenotype, together with the subcellular localization of Ci-E(z) at this stage, suggests that this protein may play important developmental roles in addition to chromatin modification by deposition of H3K27me3. This is reminiscent to what was previously described in EZH2 knock down of a cytoplasmic form of E(z) protein, characterized by independent H3K27me3 deposition and actin-polymerization defects (Roy et al., 2012; Su et al., 2005; Yamaguchi and Hung, 2014).

Later in development, we detected the presence of multinucleated cells in Ci-E(z) morphants similar to previous observation in the Drosophila E(z) mutants (Iovino et al., 2013). This phenotype might reflect a defect in cell cycle regulation and/or cytokinesis and thus might reflect a general role of E(z) that is conserved during metazoan evolution. This might be also consistent with recent studies conducted in mice, showing that PcG components play a role in DNA replication, cell cycle progression and embryonic development starting from the 2-cell stage (Posfai et al., 2012).

The early 4-cell stage embryonic defect observed through Ci-E(z) invalidation is concomitant to early effects on gene expression. More specifically, among the few genes analyzed, *Macho1*, *Tbx6c* and *ETR* were found overexpressed in morphants. These genes were respectively implicated in muscle (*Macho1* and *Tbx6c*) and nervous system (*ETR*) differentiation and their deregulation is coherent with the major phenotypic defects observed in Ci-E(z) morphants (i.e. mislocalization of muscle cells, sensory organs and neurons), and substantiate the early requirement of Ci-E(z) in normal development. Derepression of these genes might be a direct consequence of the loss of Ci-E(z) binding to their regulatory region. Since neither Ci-E(z) nor H3K27me3 are observed at high levels in the nucleus at the 4-cell stage, another possibility is that transcriptional upregulation maybe an indirect effect of the loss of the cytoplasmic form of Ci-E(z). The analysis of this putative regulatory role may thus represent an interesting area for future research. Tbx6c overexpression in morphants is totally consistent with the *in situ* hydridization realized with the MRLC2 probe since MRCL2 gene is directly controlled by Tbx6b and Tbx6c (Yagi et al., 2005) and was found highly and somehow ectopically expressed in Ci-E(z) morphants.

Concerning the phenotypic results in later development, such as muscle fate, our data could be correlated with mouse data where methylation of H3K27 by EZH2 was shown to play an essential role in the repression of muscle-specific genes and where the demethylase activity of UTX on H3K27me3 was required to initiate muscle fate (Caretti et al., 2004; Seenundun et al., 2010).

Repression of *Noto4*, involved in the terminal differentiation of notochord, in Ci-E(z) morphants at much later stages (from neurula to hatching) might account for the absence of notochord characterized both anatomically and through *in situ* hydridization using the Fibrn probe. Interestingly, Ci-Fibrn protein is necessary for the correct patterning of the neural cord (Yamada et al., 2009). The down regulation of Fibrn observed in Ci-E(z) morphants might also account for disorganization of the nervous system. In contrast to *Noto4*, an epidermal gene *Epi1* was found overexpressed at neurula and subsequent stages. For these late genes, expression changes may not necessarily reflect PRC2 function at their regulatory regions since indirect effects may as well be possible. Nevertheless, these results clearly show that major differentiation genes were affected throughout *Ciona intestinalis* embryogenesis as a consequence of Ci-E(z) invalidation. In striking contrast to all animal species studied so far, a minor involvement of *Hox* genes during *Ciona intestinalis* embryogenesis has been observed by (Ikuta et al., 2010), except minor ones for *Hox10* and *12*. In agreement with these data, *Hox12* was found derepressed in Ci-E(z) morphants (Fig. 8), indicating that the PcG-dependent gene regulation was partially conserved. However, this effect was seen in the morphants much after the regulatory effects on early genes and the loss of H3K27me3. Of note, a recent study suggested a late developmental role of *Hox1* (Sasakura et al., 2012). This opens the possibility that, despite the disruption of the cluster of *Hox* genes, a more classical PcG and TrxG role on this *Hox* gene might be effective later on during *Ciona intestinalis* metamorphosis, leading to the juvenile stage.

In conclusion, our data reveal that *Ciona intestinalis* embryonic development is tightly controlled through Ci-E(z) epigenetic regulation. Polycomb mutations were classically shown to act in embryogenesis primarily by maintaining *Hox* gene silencing. It is striking that Ci-E(z) invalidation affects development inducing much earlier and more drastic phenotypes than those reported as a consequence of perturbation of *Hox* gene expression. These results thus suggest new perspectives about the mode of action and interplay of Polycomb genes in the regulation of embryonic development and in a more general context of chordate evolution.

## MATERIALS AND METHODS

### Ethical statement

The research described herein was performed on *Ciona intestinalis*, a marine invertebrate. The study did not involve endangered or protected species, and was carried out in strict accordance with European (Directive 2010/63) legislation for the care and use of animals for scientific purposes although *Ciona intestinalis* is not included in the organisms designated by the legislation.

### Animal husbandry

Adult *Ciona intestinalis* were collected in the bay of Roscoff (Finistère, France). Oocytes and sperm were obtained by dissection of gonoducts, dechorionation was performed with 1.0% sodium thioglycolate (T0632, Sigma) and 0.05% actinase E (D4527, Sigma) (Mita-Miyazawa et al., 1985) and cross fertilization was performed in glass tubes. Embryos were reared in glass petri dishes at 18°C in 0.2 mm filtered sea water containing 100 U/ml penicillin, and 100 mg/ml streptomycin.

### Morpholino and synthetic mRNA microinjections

25-mer MOs for Ci-E(z) (#1: 5′-TTTGACTGCGTCATTTGCGTGATAT-3′; #2: 5′-CGATCTTGTAGTTTGACTGCGTCAT-3′) were ordered (Gene Tools, LLC), with target sequence containing the first methionine codon (underlined). MO was injected with Texas-Red Dextran (Invitrogen). 1 mM MO was used for microinjection. Control embryos injected with a universal control MO (5′-CCTCTTACCTCAGTTACAATTTATA-3′) did not alter gene expression. Every experiment was carried out with 20 or more embryos, leading to similar results.

For the rescue experiments, synthetic Ci-E(z) mRNA lacking the MO target sequence was designed. For *in vitro* RNA synthesis, a cDNA fragment containing the full coding sequence of Ci-E(z) (clone Unigene: VES99_N06) was subcloned into the pBluescriptRN3 vector (Lemaire et al., 1995) and used as a template for RNA synthesis with mMessage mMachine (Ambion). Concentrations of MO-Ci-E(z) and Ci-E(z) mRNA were 1 mM and 200 ng/µl, respectively. Being unable to increase the Ci-E(z) mRNA concentration, MO-E(z) ½ morphants were injected with MO-Ci-E(z) at 0.5 mM, Rescued ½ morphants were injected with MO-Ci-E(z) at 0.5 mM and Ci-E(z) mRNA at 200 ng/µl.

### Immunization procedure and antibody production

Antibody anti-Ci-E(z) was generated to a GST fusion protein corresponding to the N-terminal domain of the Ci-E(z) protein (residues 1–605; ENSCINT00000016333) (Montpellier Prorec platform, France). Purified fusion protein was used to immunize rabbits and sera were affinity-purified on immobilized Ci-E(z) fusion protein (Méjean et al., 1992).

### TUNEL staining and indirect immunofluorescence analysis

Embryos were fixed for 20 min with 3.7% formaldehyde in filtered seawater and then permeabilized for 20 min at room temperature with 0.2% Triton X-100 in TS solution (150 mM NaCl 25 mM Tris, pH 7.5).

TUNEL staining (Roche, *In situ* cell death fluorescein or rhodamin detection kit) was performed according to the manufacturer's instructions.

Fixed and permeabilized embryos were subjected to indirect immunofluorescence with rabbit anti-Ci-E(z) polyclonal antibodies (produced in our lab), rabbit anti-H3K27me3 antibodies (Millipore) or rabbit anti-acetylated tubulin antibodies (Sigma) and Phalloïdin-TRITC (Sigma) staining as described previously (Chambon et al., 2002). Appropriate secondary antibody was FITC-conjugated donkey-anti-rabbit immunoglobulins (Jackson Laboratories). Specimens were analyzed with a Leica TCS-SPE laser confocal microscope (Montpellier RIO Imaging platform, France).

### Acid precipitation of histone proteins and immunoblotting

Histone extraction was prepared essentially as described in (Bredfeldt et al., 2010). Briefly, 15–30 mg of staged larvae were resuspended in 150 µl RSB buffer (10 mM Tris-HCl, pH 7.4; 10 mM NaCl; 3 mM MgCl_2_; Complete Protease Inhibitor Cocktail) with 0.5% Nonidet P-40, homogenized using a Dounce homogenizer and incubated on ice for 10 min. Lysates were then centrifuged for 5 min at 9,000 ***g*** at 4°C. The pellet was resuspended in equal volumes (75 µl) of 5 mM MgCl_2_ and 0.8 M HCl, passed 4 times through a 25G5/8 gauge needle, and incubated on ice for 1 h. The solution of histone proteins was centrifuged at 16,000 ***g*** for 10 min at 4°C. Supernatant was transferred to a new tube, and histones were precipitated with a 25% of trichloroacetic acid (TCA) solution dissolved in deionized water. Precipitated histones were collected by centrifugation for 20 min at 16,000 ***g*** at 4°C. The resultant pellet was washed with ice-cold acetone, dried, and subsequently resuspended with deionized water (30 µl) and 2 M Tris-HCl, pH 8.0 (0.3 µl). Histone proteins (4 µl) were resolved on 20% SDS-PAGE gel and Coomassie stain was used to correct for variations in histone H3 loading.

Then, histones were separated by SDS-PAGE, transferred to a PVDF membrane (Immobilon; Millipore), and changes in histone methylation relative to total histone H3 were investigated by western blot analysis. Membranes were probed with rabbit anti-histone H3 (1:5000; ab1791, Abcam) and anti-H3K27me3 (1:5000; 07-449, Millipore) in 5% BSA, Tris-buffered saline with 0.1% Tween 20, for 2 h at room temperature, followed by incubation with horseradish peroxydase-conjugated donkey anti-rabbit secondary antibody (1:10,000; Jackson ImmunoResearch). The blots were visualized by an enhanced chemiluminescence detection system (Pierce).

### Light and transmission electron microscopy (TEM)

Embryos were fixed in 2.5% glutaraldehyde in 0.2 M cacodylate buffer (pH 7.2) and post-fixed in 1% osmium tetroxide in 0.45 M cacodylate buffer (pH 7.2). The fixed material was dehydrated in a graded alcohol series and embedded in Epon 812. For light microscopy, semi-thin sections were stained with Toluidine Blue and observed on a Reichert microscope equipped with Nomarski optics. For TEM, ultra-thin sections were classically contrasted with uranyl acetate and lead citrate (Reynolds, 1963), and observed with a Jeol 1200X transmission electron microscope (UM2 TEM platform, France).

### mRNA isolation and RT-qPCR

Fertilized and microinjected eggs were collected at different stages of development from t=0 h to t=18 h after fertilization. Total RNA was isolated with RNeasy kit according to the supplier's instructions (QIAGEN). Reverse transcription was performed on equal input mRNA by Superscript II reverse transcriptase (Invitrogen) with an oligodT primer. qPCR was realized on a Light Cycler 480 with the SYBR Green I Master kit (Roche) (qPHD UM2/GenomiX Platform, Montpellier - France) and monitored using S26 control primers. Sets of primers designed to amplify each selected gene are listed in Table 2.

**Table 2.**
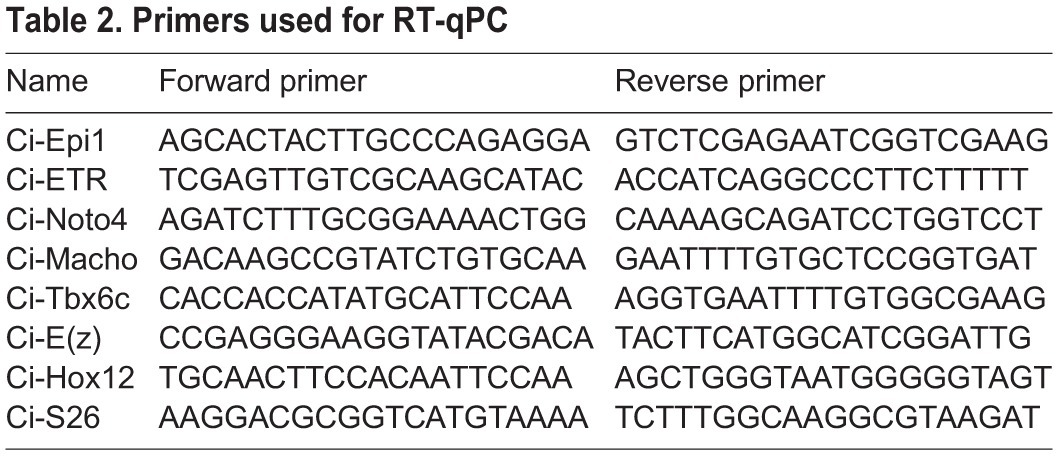
**Primers used for RT-qPC**

### *In situ* hybridization

Linear cDNA template for RNA probes was created by RT-PCR using T3 forward and T7 reverse primers to amplify cDNA from Ci-Fibrn (KH2012:KH.C1.832) (Takahashi et al., 1999) and Ci-MRLC2 (KH2012:KH.C8.859) (Ikuta et al., 2010). PCR products were sequenced and purified (High Pure PCR Product Purification Kit, Roche; 11732668001). Purified PCR product (300 ng) was used as template for the DIG RNA Labeling Kit (Roche; 11277073910) and labelled RNA probes were then purified with Microspin G-50 (Dutsher; 27-5330-01).

Embryos were fixed overnight at 4°C (0.1 M MOPS pH 7.5, 0.5 M NaCl, 4% PFA) and dehydrated through a PBS/ethanol series before storing at −20°C in 75% ethanol/PBS.

Embryos were rehydrated through an ethanol/PBT (PBT: 1× PBS, 0.1% Tween) series, treated with proteinase K (4 µg/ml in PBT, 25 min, 37°C), washed with glycine (2 mg/ml in PBT) then fixed again (4% PFA in PBT, 1 h at RT). Embryos were pretreated with hybridization buffer (1× Denhardt's, 6× SSC, 50% Formamide, 0.1% Tween, 50 µg/ml tRNA) 10 min at RT followed by 1 h at 55°C. Heat denatured probe (100 ng) was then added and embryos were hybridized for approximately 20 h at 55°C. After hybridization, embryos were washed at 55°C: 2×20 min with WB1 (50% Formamide, 5× SSC, 0.1% SDS), 2×20 min with 1:1 WB1:WB2, 2×20 min with WB2 (50% Formamide, 2× SSC, 0.1% Tween20) and 2×20 min with WB3 (2× SSC, 0.1% Tween). Embryos were preblocked (0.1 M Tris, pH 7.5, 0.15 M NaCl, 0.5% BSA, 2% FCS, 1 h at RT) then stained (2 h at RT and after overnight at 4°C) with anti-DIG-AP Fab fragments (1:2000 dilution; Roche, 11093274910). Embryos were washed with PBT (6×10 min at RT) and 3×10 min with TMN buffer (100 mM NaCl, 50 mM MgCl2, 100 mM Tris-HCl pH 8, 0.1% Tween) then stained with NBT-BCIP (Roche, tablets 11697471001) for 10 min to 2 h in the dark, depending upon the probe used. Embryos were washed in PBT and post-fixed with 4% PFA in PBT, 1 h at RT. They are cleared through 50% glycerol/PBT and mounted to coverslips in Mowiol mounting medium. Embryos were imaged on a Leitz Diaplan microscope using a Leica DC300F camera.

### Database analysis

E(z) protein sequences from *D. melanogaster* and *H. sapiens* were found on NCBI (http://www.ncbi.nlm.nih.gov/). For each species, when several proteins were available for the same gene the longest was retained. Each selected sequence was then used to conduct TBLASTN searches in *Ciona intestinalis* genomic (http://genome.jgi.doe.gov/Cioin2.home.html) and cDNA (http://ghost.zool.kyoto-u.ac.jp/blast_kh.html) databases. Among hit sequences, we selectively kept those that were indifferently found with both reference species. As expected, the closer the reference species is to *Ciona intestinalis*, the more the e-value is significant (data not shown). This step gave clusters of sequences that we validated with Aniseed website (http://www.aniseed.cnrs.fr/) and for which we searched for functional domains required for activity with a CD-search on NCBI (http://www.ncbi.nlm.nih.gov/cdd) against CDD-43212 PSSMs database.

## Supplementary Material

Supplementary information
