# Supplementary material for: Enhancer of zeste acts as a major developmental regulator of Ciona intestinalis embryogenesis
Source: Biol Open. 2015 Aug 14;4(9):1109–21. doi: 10.1242/bio.010835 (PMC4582116; doi:10.1242/bio.010835)
Supplement: Supplementary information [file supp_4_9_1109__index.html]

Enhancer of zeste acts as a major developmental regulator of Ciona intestinalis embryogenesis — Supplementary information 

# Enhancer of zeste acts as a major developmental regulator of *Ciona intestinalis* embryogenesis

## BIO010835 Supplementary information

**Files in this Data Supplement:**

- Supplementary information
