# Supplementary material for: Enhancer of zeste acts as a major developmental regulator of Ciona intestinalis embryogenesis
Source: Biol Open. 2015 Aug 14;4(9):1109–21. doi: 10.1242/bio.010835 (PMC4582116; doi:10.1242/bio.010835)
Supplement: Supplementary information [file supp_bio.010835_BIO010835supp.pdf]

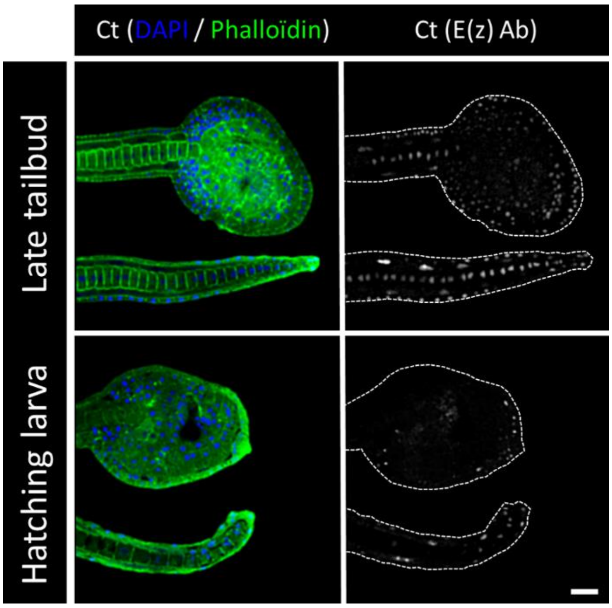

Fig. S1. Localization of Ci-E(z) protein in control embryos at late tailbud and hatching stages. Ci-E(z) protein, actin (Phalloïdin, green) and DNA (DAPI, blue) were localized by triple labeling in *Ciona intestinalis* embryos by confocal microscopy. At the right of each merge (actin/DNA) the corresponding Ci-E(z) image is shown with the cell contours drawn in grey. Scale bar: 25 µm.

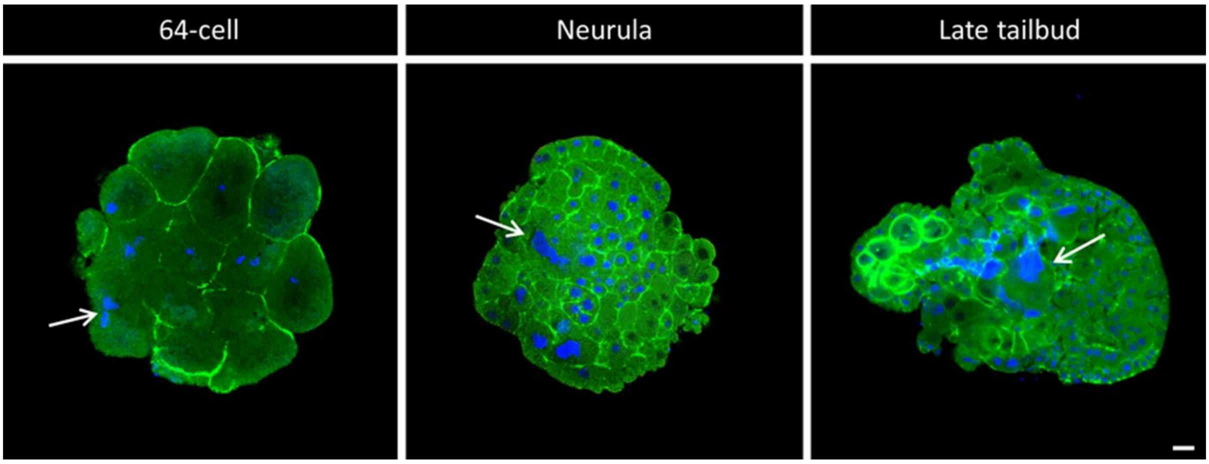

Fig. S2. Presence of cytokinesis defects all along embryogenesis. Ci-E(z) morphants from different stages of embryogenesis (64-cell, neurula and late tailbud stages) were collected and analyzed by indirect immunofluorescence with double labeling: actin (Phalloïdin, green) and DNA (DAPI, blue). Arrows point out multinucleated cells. Scale bar: 25 µm.

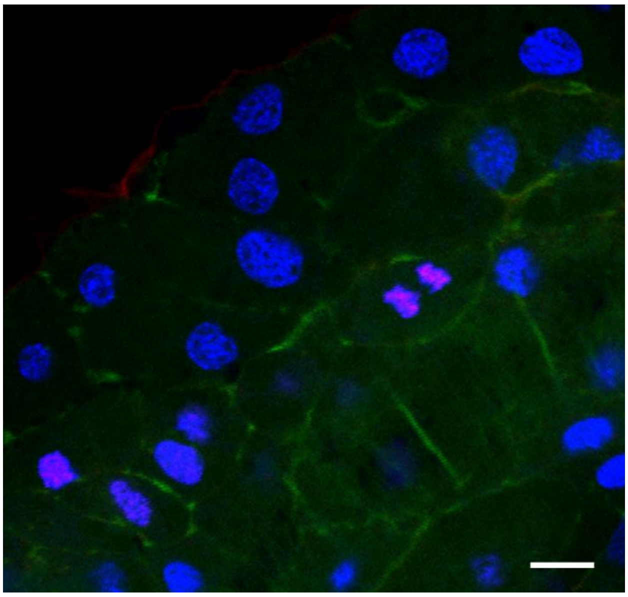

Fig. S3. H3K27me3 detection during mitosis in control embryos. H3K27me3 (red), actin (Phalloïdin, green) and DNA (DAPI, blue) triple labeling of *Ciona intestinalis* embryos at middle tailbud stage. Scale bar: 8 µm.

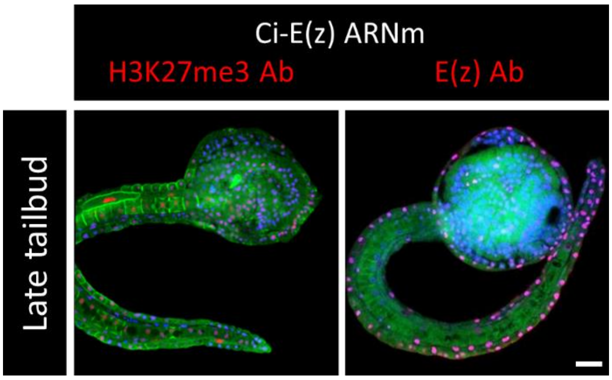

Fig. S4. Ci-E(z) and H3K27me3 detection in mRNA control from rescue experiments. Phenotype of embryos obtained after microinjection of Ci-E(z) mRNA alone and collected at late tailbud stage of development. A triple labeling was performed using: actin (Phalloïdin, green), DNA (DAPI, blue) and antibodies against Ci-E(z) protein or H3K27me3 (red). Scale bar: 25 µm.
